# Advancing pharmacological treatments for opioid use disorder (ADaPT-OUD): protocol for testing a novel strategy to improve implementation of medication-assisted treatment for veterans with opioid use disorders in low-performing facilities

**DOI:** 10.1186/s13722-018-0127-z

**Published:** 2018-12-13

**Authors:** Hildi Hagedorn, Marie Kenny, Adam J. Gordon, Princess E. Ackland, Siamak Noorbaloochi, Wei Yu, Alex H. S. Harris

**Affiliations:** 1 Center for Chronic Disease Outcomes Research, Minneapolis Veterans Affairs Health Care System, 1 Veterans Drive, Mail Code#152, Minneapolis, MN 55417 USA; 2 Department of Psychiatry, University of Minnesota School of Medicine, Minneapolis, USA; 3 Informatics, Decision-Enhancement, and Analytic Sciences Center (IDEAS 2.0), VA Salt Lake City Health Care System, 500 Foothill Drive, Salt Lake City, UT 84148 USA; 4 Program for Addiction Research, Clinical Care, Knowledge, and Advocacy (PARCKA), Division of Epidemiology, Department of Internal Medicine, University of Utah School of Medicine, 30 N 1900 E, Salt Lake City, UT 84132 USA; 5 Center for Chronic Disease Outcomes Research, Minneapolis Veterans Affairs Health Care System, South. Bldg. 9 Rm#316, Mail Code#152, Minneapolis, MN 55417 USA; 6 Department of Medicine, University of Minnesota, Minneapolis, USA; 7 Center for Chronic Disease Outcomes Research, Minneapolis Veterans Affairs Health Care System, South. Bldg. 9 Rm#219, Mail Code#152, Minneapolis, MN 55417 USA; 8 Health Economics Research Center, VA Palo Alto Health Care System, 795 Willow Road (152 MPD), Menlo Park, CA 94025 USA; 9 Center for Health Policy and Center for Primary Care and Outcomes Research, Stanford University, Stanford, USA; 10 Center for Innovation to Implementation, Palo Alto Health Care System, 795 Willow Rd, MC152, Palo Alto, CA 94304 USA; 11 Department of Surgery, Stanford University, Stanford, USA

**Keywords:** Substance use disorder, Opioid agonist therapy, Implementation science

## Abstract

**Background:**

In the US, emergency room visits and overdoses related to prescription opioids have soared and the rates of illicit opioid use, including heroin and fentanyl, are increasing. Opioid use disorder (OUD) is associated with higher morbidity and mortality, higher HIV and HCV infection rates, and criminal behavior. Opioid agonist therapy (OAT; methadone and buprenorphine) is proven to be effective in treating OUD and decreasing its negative consequences. While the efficacy of OAT has been established, too few providers prescribe OAT to patients with OUD due to patient, provider, or system factors. While the Veterans Health Administration (VHA) has made great strides in OAT implementation, national treatment rates remain low (35% of patients with OUD) and several facilities continue to have much lower prescribing rates.

**Methods:**

Eight VA sites with low baseline prescribing rates (lowest quartile, < 21%) were randomly selected from the 35 low prescribing sites to receive an intensive external facilitation implementation intervention to increase OAT prescribing rates. The intervention includes a site-specific developmental evaluation, a kick-off site visit, and 12 months of ongoing facilitation. The developmental evaluation includes qualitative interviews with patients, substance use disorders clinic staff, and primary care and general mental health leadership to assess site-level barriers. The site visit includes: (1) a review of site-specific barriers and potential implementation strategies; (2) instruction on using available dashboards to track prescribing rates and identify actionable patients; and (3) education on OAT, including, if requested, buprenorphine certification training for prescribers. On-going facilitation consists of monthly conference calls with individual site teams and expert clinical consultation. The primary outcomes is the proportion of Veterans with OUD initiating and sustaining OAT, with intervention sites expected to have larger increases in prescribing compared to control sites. Final qualitative interviews and a cost assessment will inform future implementation efforts.

**Discussion:**

This project will examine and respond to barriers encountered in low prescribing VHA clinics allowing refinement of an intervention to enhance access to medication treatment for OUD in additional facilities.

## Background

The availability of prescription opioid pain medications has skyrocketed in the United States over the past 20 years. With greater availability comes an increase in the negative consequences related to prescription opioid use. Emergency visits related to non-medical use of prescribed opioids increased 117% from 2005 to 2011 [1] and overdose deaths related to opioids nearly quadrupled between 2000 and 2014 [2]. In addition, there has been a corresponding increase in the use of heroin. Individuals reporting past year heroin use has nearly doubled from 2005 to 2012 [3]. Opioid use disorders (OUD) have been estimated to have a total societal cost of $55.7 billion, with $25 billion of those costs related to health care [4], and patients with OUD incurring significantly higher annual direct health care costs compared to those without OUD diagnoses [5–8]. Among Veterans receiving care at Veterans Health Administration (VHA) facilities in 2017, more than 69,000 had a diagnosis of OUD, a number that has doubled in the last 4 years [9].

Opioid agonist therapy (OAT) with methadone or buprenorphine is the gold-standard treatment for OUD. Methadone treatment has been associated with improved retention in treatment, and with reductions in IV drug use, criminal activity, HIV risk behaviors and mortality [10–12]. However, methadone can only be delivered in highly regulated Opioid Treatment Programs. Buprenorphine, introduced in the United States in 2002, is a partial opioid agonist. Numerous outpatient studies have shown that sublingual buprenorphine maintenance treatment is equally effective as moderate doses of methadone [13–19]. After completing 8 h of mandatory training and obtaining a waiver from the US Drug Enforcement Agency, physicians can prescribe buprenorphine in an office-based setting, including primary care, thus potentially increasing access to treatment. Recently, other practitioners with prescriptive authority (e.g., advanced practice nurses, physician assistants) can obtain a waiver to prescribe with 24 h of training. When OAT is contraindicated or not acceptable to the patient, antagonist medication (naltrexone) can be considered.

This paper describes the protocol of a project that focuses primarily on implementation of buprenorphine due to the lower regulatory burden of prescribing buprenorphine compared to methadone and the higher level of evidence for effectiveness compared to naltrexone. The project will also encourage implementation of injectable naltrexone for cases where buprenorphine is not a viable or acceptable alternative for a particular patient. Increasing implementation of OAT is imperative because, despite the scope of the current problem, it is grossly underutilized. Of the 2.1 million people with OUD in the United States, it is estimated that only 20% receive any form of treatment [20]. Only half of private treatment programs have adopted OAT and in those that do offer OAT, only one third of patients receive it [21].

VHA policy is fully supportive of OAT for OUD. The Uniform Mental Health Services Benefits Package states: “Pharmacotherapy with approved, appropriately regulated opioid agonists (buprenorphine or methadone) must be available to all patients diagnosed with opioid dependence for whom it is indicated and for whom there are no medical contraindications” and “when agonist treatment is contraindicated or not acceptable to the patient, antagonist medication (e.g., naltrexone) needs to be available and considered for use when needed”. Models of implementation of buprenorphine in several settings within the VHA (e.g., primary care, mental health care, Opioid Treatment Programs, addiction specialty care) exist and have been described in the literature [22, 23]. Implementation of OAT in VHA has primarily been promoted by the Buprenorphine in the VHA (BIV) Initiative which was established in 2007 by the Center of Excellence in Substance Abuse Treatment and Education (CESATE). The BIV has been instrumental in enhancing Veteran access to buprenorphine in the VA by providing buprenorphine consult and mentoring services to VA clinicians nationwide with a variety of resources including monthly newsletters, monthly webinar in-services, a voluntary VA buprenorphine provider listing, resource guides, and SharePoint website information repositories [24]. More recently, the Office of Mental Health and Suicide Prevention (OMHSP) has begun monitoring progress toward implementation of Uniform Mental Health Services Benefits Package requirements, including the rate of pharmacotherapy for OUD, through the Mental Health Information System Dashboard (MHIS). Among many other metrics, the MHIS provides the facility-level percentage of patients with OUD receiving medication treatments for OUD. Facilities can view their own percentage, that of other VHA facilities in their region and throughout the nation as well as the national VHA average in order to evaluate their performance on implementation of medication treatments for OUD.

Through these combined efforts, VA has greatly expanded access to buprenorphine from only 324 Veterans receiving buprenorphine in Fiscal Year 2004 to 15,135 Veterans receiving buprenorphine in Fiscal Year 2017. Despite these efforts and the rapid growth in prescribing, only 35% of VHA patients with OUD diagnoses received medication treatment. Most concerning is that this rate is highly variable across VHA facilities with some facilities remaining that do not provide any access to medication treatments.

This project has the following aims:

1. Test the effectiveness of intensive external facilitation on: (a) the proportion of patients with OUD initiating OAT, and (b) the proportion who engage in long-term OAT (a minimum of 3 months) at 8 randomly selected low-performing VA sites compared to the remaining 27 low-performing sites receiving implementation as usual.
2. Use developmental, implementation-focused, progress-focused, and interpretative formative evaluation techniques [25] to: (a) finalize the intervention, (b) assess fidelity to the implementation plan, (c) track progress toward implementation goals, and (d) enhance interpretation of quantitative results.
3. Assess the cost and budget impact of the implementation intervention.

## Methods/design

Guided by the integrated Promoting Action on Research Implementation in Health Services (i-PARIHS) framework [26, 27], the objective of this project is to test whether enhancing ongoing implementation efforts with intensive external facilitation can speed implementation of OAT compared to continued “implementation as usual” in low-adopting VHA facilities. “Implementation as usual”, as described above, consists of policy mandates, guideline recommendations, and consultation, webinars and dashboards available to sites that seek out these resources. While OAT itself has been shown to be cost effective [28, 29], the project will also document the costs and budget impact of the intensive external facilitation intervention.

This study will provide essential information to our partners in VHA Office of Mental Health and Suicide Prevention (OMHSP) regarding the effectiveness and budget impact of using intensive external facilitation as a strategy to enhance implementation of OAT in low-performing sites and may inform the design of efforts to implement other evidence-based practices in low-performing sites both within and outside of VHA.

## Site selection

Criteria for site recruitment included a current facility-level prescribing rate in the lowest quartile of all VHA facilities (< 21% of patients with OUD diagnoses receiving medication treatment) as identified through the MHIS dashboard. As described above, the MHIS includes the percent of patients with an OUD diagnosis receiving OAT at each VHA facility, allowing comparisons to other VHA facilities and the VHA national average. At the time of site selection, there were 35 VHA facilities that met this requirement. These 35 facilities were stratified based on their prescribing rate (low < 15%, high = 15–21%) and the number of “actionable patients” at the facility (low < 472, high = 472+; see Table 1). “Actionable patients” indicate the number of patients who have a diagnosis of OUD but do not have an OAT prescription. The number of actionable patients at a facility was determined using the Psychotropic Drug Safety Initiative (PDSI) dashboard which is a panel management tool that uses real time data to assist local facilities in identifying actionable patients for review. Two sites were randomly selected from each block. The purpose of this stratification was to ensure representativeness of the variability that exists within the low prescribing group. Participation in the initiative was voluntary. Sites that were not interested in participating were replaced with a site with a similar baseline prescribing rate and similar number of actionable patients from the remaining pool of low prescribing sites. Low prescribing sites that were not selected for the intervention served as control comparison sites. The control sites did not have the option of declining participation. The research team had no contact with the control sites. Administrative data was used to monitor prescribing rate at control sites.

**Table 1:** Criteria for stratification of low-performing sites based on percent of patients with OUD receiving medication treatment and number of actionable patients

Number of actionable patients	Percent of patients with OUD receiving medication treatment	
	Low	High
Low	≤ 15%	> 15 ≤ 21%
	< 472 patients	< 472 patients
High	≤ 15%	> 15 ≤ 21%
	≥ 472 patients	≥ 472 patients

The primary outcome measure is the facility-level prescribing rate. This is the percentage of patients with an OUD diagnosis on their chart who are receiving medication treatment for their OUD. Each VHA facility offers both inpatient and outpatient services and the facility-level prescribing rate does not distinguish between patients who received medications in an inpatient setting versus an outpatient setting. Facilities could increase their prescribing rate with a variety of strategies including increasing outpatient prescribing or increasing inpatient prescribing with linkage to outpatient follow-up and maintenance of medications. The facility-level identified strategies are determined by the external facilitation intervention as described below. Qualitative formative evaluation data documents the settings that facilities focus on for their implementation strategies and whether and how successful their implementation efforts are in those settings.

## The intensive external facilitation intervention

### Stakeholder interviewing

One of the key roles of a facilitator is to assess local conditions in order to determine where key barriers to implementation lie, e.g., Do local providers and patients view OAT for OUD as an acceptable treatment? Do providers feel that they do not have the skills necessary to successfully provide OAT for OUD? Do they not have enough staff with prescribing privileges available in the clinic? The facilitator(s) then use this information to tailor implementation strategies to the most predominant local barriers. Assessing local conditions also allows facilitators to identify local individuals that can serve as clinical champions based on their interest and/or experience in the area. In order to assess local conditions and identify potential local champions, we began our intervention with qualitative interviewing guided by the i-PARIHS framework which identifies key features of the Innovation, Recipients, and Context constructs that need to be considered when assessing for barriers to implementation. Interviews are being conducted by phone with approximately 10 individuals from each site.

We are starting with phone interviews of the substance use disorders specialty clinic medical directors and program coordinators. With the assistance of the medical director and/or the program coordinator, we identify at least one psychiatrist, nurse, pharmacist and case manager to be interviewed. While case managers may not be directly involved with the prescribing of OAT, their attitudes toward the treatment can impact both other providers’ and patients’ willingness to consider OAT as a viable treatment option. We also interview the local primary care chief and mental health chief. Because most physicians with the necessary training and waiver to prescribe buprenorphine have a maximum caseload of 30 or 100 patients, capacity within the substance use disorder (SUD) specialty clinic may be an issue for sites with very large numbers of actionable patients. Therefore, examining the potential for developing capacity for primary care providers and general mental health psychiatrists to continue prescribing once a patient has started treatment may be a vital option to investigate. We are also asking the local SUD program staff at each site to assist in recruiting two patients with OUD diagnoses currently in recovery who would be willing to participate in an interview to gain an understanding of patient perspectives on available local OUD treatment and attitudes toward OAT. This will give us a total patient sample of 16 interviews.

Data from the interviews are analyzed rapidly so that they inform the specific implementation strategies to be employed at each site. Project staff read transcripts of all interviews from a site and take detailed notes on identified barriers to implementation and patient perspectives. Once all interviews from a particular site have been reviewed, the qualitative core (Hagedorn, Ackland, Kenny, Valenstein-Mah) meet to review the notes from that particular site and generate a comprehensive list of identified barriers and perspectives. A report is then generated for each site detailing barriers along with potential implementation strategies targeted to each major barrier. This report is used during the next phase of the intervention, the facilitators’ site visit, where one of the first activities is to review the report and solicit input from the local team as to the feasibility of the plan. While the specific barriers and strategies will vary by site, the intervention that is being tested in this project is intensive external facilitation which involves a process of identifying local barriers and developing specific site-level intervention plans based on identified barriers.

### Facilitators’ site visit

Once the interviews and summary of barriers are complete for a site, the facilitators schedule a 1- to 2-day site visit at each of the 8 intervention sites. Potential local champions identified through the interview process are contacted to solicit assistance with identifying additional staff to serve on the local implementation team and assist with scheduling and planning for the site-visit. The length of the site visit depends on the educational needs of the individual site. Some components of the site visit are consistent across all intervention sites. Consistent components include meeting with the local team to: (1) review the local site report on barriers and potential strategies, (2) educate the local team about identified patient perspectives and available resources, and (3) assist the local team in developing an action plan to implement identified strategies. The facilitators review the local site report with the local team to ensure that they are in agreement with the major identified barriers and that no key barriers were missed. They also review the proposed strategies to ensure that the local team agrees with the feasibility of the identified strategies and solicit suggestions for additional strategies that may have been overlooked. The facilitators educate the local team about the VA/Department of Defense Clinical Practice Guidelines for the Management of Substance Use Disorders as well as existing Buprenorphine in the VA initiative (BIV) resources that they can access including national email lists, monthly webinar in-services, resource guides and SharePoint information repositories. They educate the local team about how to access existing dashboards (Mental Health Information Services, Psychotropic Drug Safety Initiative) and how to use them to both monitor facility performance of the percent of patients with an OUD diagnosis receiving OAT and identify actionable patients. Finally, they assist the local team in development of an action plan for implementing identified strategies with the assistance of the facilitation team. The action plan details specific actions along with timelines for completion of actions and responsible parties (which may be local team members or facilitation team members).

The remaining components of the site visit are educational and are tailored to meet the needs of the individual sites based on the site report and discussion in advance with the local team regarding the site visit agenda. Trainings that the facilitators offer to present to provider groups include:

1. The 4-h face-to-face portion of the 8-h Providers’ Clinical Support System for Medication Assisted Treatment (PCSS-MAT) “half and half” training. This face-to-face training, in combination with 4 h of on-line education for physicians and 20 h of on-line training for advance practice nurses and physician assistants, allows providers to qualify for a waiver from the special registration requirements in the Controlled Substances Act for the provision of OAT. This can be offered to SUD specialty care, general mental health and/or primary care providers as needed.
2. General overview of OAT treatment for OUD with heavy emphasis on treatment and cost effectiveness compared to other treatment modalities (psychosocial/behavioral only, detoxification) for all SUD treatment staff. This training is designed primarily to counteract negative attitudes toward OAT.
3. Presentation of strategies for integrating office-based buprenorphine treatment into current SUD specialty clinic structures, e.g., how to set up induction monitoring and structures for monitoring patients over time. This training can be presented as a stand-alone for SUD specialty clinic staff or can be combined with #2 above if “buy-in” needs to be established first.
4. General overview of the scope of the OUD epidemic, negative consequences of OUDs, overview and effectiveness of OAT for OUD, and models for integrating ongoing OAT into primary care and/or general mental health settings. This training is designed primarily to educate primary care and/or general mental health providers and persuade them to complete mandatory training and become waivered providers to assist with capacity building.

For all site visit activities, facilitators record the length of the meeting, the number of attendees and their job classification. This information will be used to document the cost of the intervention.

### Ongoing facilitation

The exact tasks performed by facilitators are driven by the local assessment of implementation barriers and the local action plan. At a minimum, ongoing facilitation consists of 12 months of:

1. Monthly conference calls with each of the 8 intervention sites and the facilitation team. The monthly conference calls allow the facilitation team to review progress with each individual site, identify new or persistent implementation barriers, suggest potential strategies, and identify local site needs that facilitators can assist with. The facilitation team is also available on an as-needed basis through email and phone contact to assist with implementation issues that arise between regularly scheduled calls.
2. Monthly all-site conference calls: The monthly all-site conference call allows for community building and allows sites to share barriers and problem-solve potential strategies. An email group for the community allows interaction on an as-needed basis between monthly calls.
3. Availability of expert consultation services: expert consultation services are provided by a research team member who is a nationally recognized expert in office-based buprenorphine treatment (Gordon). Consultation is available on demand via email and telephone or tele-conferencing to assist site physicians with initial buprenorphine inductions in real time.

Based on prior projects led by the study team, external facilitation can include meeting with local Pharmacy and Therapeutics boards to request lifting local prescribing barriers for targeted pharmacological agents, developing educational materials such as pocket cards and one-page evidence summaries, and modifying local progress reports for site teams to share with local leadership.

Contact logs were created for each intervention site to document on-going facilitation activities. Each regularly scheduled and ad-hoc contact with the facilitators is documented including the time spent on the activity and the number of local participants and their job classifications. The facilitators also document time that they spend on additional facilitation-related activities such as procuring or developing requested resources. This information will be used to document the cost of the intervention.

## Data analysis

### Aim 1: Effectiveness

The study includes three distinct time periods. Pre-implementation includes the six months prior to the site visit (Phase 1). Implementation includes the 12-month period of active implementation following the site visit (Phase 2). Sustainment includes the 9-month period following the end of active implementation (Phase 3).

#### **Hypothesis 1**

For intensive external facilitation sites, the proportion of Veterans with OUD diagnoses receiving OAT will increase significantly from pre-implementation (Phase 1) to the end of implementation (Phase 2) and this increase will be significantly greater than for implementation as usual sites.

#### **Hypothesis 2**

For intensive external facilitation sites, the proportion of Veterans with OUD who persist with OAT for 90 days or greater will increase significantly from pre-implementation (Phase 1) to the end of implementation (Phase 2) and this increase will be significantly greater than for implementation as usual sites.

#### **Hypothesis 3**

Increases in receipt of and persistence on OAT for intensive external facilitation sites will be maintained during the sustainment period. Hypothesis 3 mirrors hypothesis 1 and 2 but compares the two outcomes (proportion of Veterans with OUD receiving OAT and proportion of Veterans sustaining OUD) from pre-implementation (Phase 1) to the end of the sustainment period (Phase 3).

Since our change measures (proportion of Veterans with OUD receiving OAT and sustaining OAT over 90 days) are recorded from all the eligible facilities through their usual recording activities, data can be collected for all the patients of all the eligible facilities. We will use all these sites and all of their corresponding OUD patients in our study. However, the source of sampling variation is through random site selection for the implementation intervention.

We will record the two primary outcomes for each patient with an OUD diagnosis: (1) whether s/he has received OAT, and (2) whether s/he has persisted with OAT for at least 90 days. These primary measures with be collected at the end of the pre-implementation phase for all patients with an OUD diagnosis during the 6-month pre-implementation phase, at the end of the implementation phase for all patients with a diagnosis of OUD during the 12-month implementation phase, and at the end of the sustainment phase for all patients with a diagnosis of OUD during the 9-month sustainability phase. Also, administratively available patient, provider and facility covariates will be collected for all the patients in all the 35 sites. Simple summary statistics, estimates and confidence intervals will be constructed. The classical repeated measure design will be modelled through a stratified/generalized linear logistic mixed model incorporating the possible site and provider dependence and clustering into the analysis.

### Aim 2: Formative evaluation

Finalization of the intervention is informed by the rapid analysis of the pre-implementation staff and leadership interviews. Fidelity to the implementation plan is monitored using contact logs created for each intervention site to document on-going facilitation activities. These logs will provide information on the “dose” of facilitation received at each site, the number of local implementation team members involved and their roles, and the specific facilitation strategies employed (e.g., education, technical support, resource identification/development, consultation). Progress toward implementation goals is tracked through quarterly feedback reports of prescribing rate, actionable patients and waivered providers. At the end of the implementation process, all of these sources, along with post-intervention provider and leadership interviews, will be used to enhance interpretation of quantitative results. Post-intervention interviews will also be conducted to determine whether barriers identified during the pre-implementation interviews were successfully addressed and which implementation strategies were most successful, acceptable and feasible. Following the implementation phase, pre- and post- implementation qualitative interviews will be coded and analyzed guided by the i-PARIHS framework to examine the impact of evidence, context and facilitation constructs on the implementation process and outcomes.

### Aim 3: Cost assessment

The budget impact analysis for the proposed intervention will be conducted from the VHA’s perspective by Dr. Wei Yu and his team. Dr. Yu, is a Health Economist who has been faculty at the VA Health Economics Resource Center (HERC) conducting cost analyses using VHA administrative data for over 10 years. For each facility, we will estimate three cost components: (1) intervention cost, (2) treatment cost and (3) downstream cost.

*Intervention cost*: The intervention in this study is the implementation strategy of intensive external facilitation. We will identify costs related to the intensive external facilitation by a micro-costing method. We will document every action taken during the three steps of intervention: stakeholder interviews, facilitators’ site visit, and ongoing facilitation. Costs associated with staff participation in interviews, site-visit meetings, education workshops and conference calls will be measured by meeting time and staff average salary. The cost associated with facilitators and consultants will be measured separately as it may not affect the budget of local sites. We will use the VA Managerial Cost Accounting System (MCA) Account-Level Budgeter Cost Center (ALBCC) datasets to identify costs for staff time at average levels in each category of staff involved in those activities.

*Treatment cost*: We will estimate the incremental cost of increasing buprenorphine treatment following the proposed implementation intervention. We will first estimate an average treatment cost for buprenorphine. Then, we will estimate the net increase in buprenorphine use by a difference in difference method (i.e., the difference between the pre-post difference and the intervention-control difference). We will identify healthcare costs from MCA national data extract (NDE), including inpatient, outpatient and pharmacy data.

*Downstream cost*: Increasing buprenorphine utilization is likely to decrease emergency department visits and emergency hospitalizations associated with overdose and other complications of OUD. We will analyze changes in health care utilization related to OUD following the proposed intervention. First, we will estimate the difference of an average annual cost of all healthcare utilization excluding OAT between those who received buprenorphine and those who did not receive any OAT, respectively. Then, we will estimate the net increase of buprenorphine use following the intensive external facilitation using the difference-in-difference method discussed in the treatment cost analysis above. We will then estimate the downstream cost by multiplying the average cost difference estimated in step 1 by the net increase of buprenorphine treatment following the implementation intervention. We will separate healthcare related to OUD and care for other health conditions.

## Discussion

This project tests intensive external facilitation to promote implementation of the most effective, evidence-based treatment for OUD in low-adopting VHA facilities that, to date, have not responded to operations-level implementation efforts. If successful, this project will detail an effective intervention to promote uptake of OAT for OUD in low-performing facilities and provide an intervention model which could be used in the future to assist low-performing facilities in implementing other evidence-based treatments. The cost assessment will allow operations partners to evaluate the cost relative to the impact of the intervention in determining whether broader dissemination is appropriate.

Although the specific barriers encountered and strategies employed at each site will likely be in some ways unique, through the formative evaluation process, we intend to identify common barriers and effective strategies that other facilities or external facilitators could use to assist in evaluating their own situation and identifying potential solutions. Because of our stratification process, our intervention sample includes both highly rural and highly urban sites which have vastly different issues related to either starting up OUD medication treatment programs from scratch or expanding on existing robust medication treatment programs that are not keeping up with the large demand placed on them. Our intent is to create a “handbook” that describes prototypical sites, the common barriers they face, and effective strategies that they employed so that additional facilities (or external facilitators working with them) could speed the process of identifying possible solutions to their implementation issues. While this intervention is limited to VHA facilities, lessons learned could be applied to other clinical settings. Similar barriers and challenges overcome by facilities in this study may exist in clinics outside the VHA health care system. The successful strategies could aid clinicians in their own expansion efforts.

We anticipate some challenges in this intervention. For example, staff turnover in any intervention of this type makes it difficult to gain traction. We plan to address that by continually engaging staff with monthly facilitation calls, newsletters and all site forums where staff from different sites can share successes and challenges. We will also provide feedback reports with prescribing rates, number of prescribers and number of actionable patients comparing each site to the other sites in the intervention to serve as a motivator.

The cost impact of adopting an intervention will vary by facility, due to differences in staff/physician capacity, management skill, economy of scale, etc. Facilities with higher implementation costs are likely to have lower adoption rates. Therefore, we need to understand the types and variations of costs when conducting a budget impact analysis.

With increasing efforts in quality improvement at VHA, adequate funding for low-performing facilities is needed, otherwise, we may crowd out other high value services.

## Abbreviations

ADS: academic detailing service
ALBCC: Account-Level Budgeter Cost Center
BIV: Buprenorphine Initiative in the VA
CESATE: Center of Excellence in Substance Abuse Treatment and Education
HCV: Hepatitis C virus
HERC: Health Economic Resource Center
HIV: human immunodeficiency virus
i-PARIHS: Integrated Promoting Action on Research Implementation in Health Services
MCA: managerial cost accounting
MHIS: Mental Health Information Service
NDE: national data extract
OAT: opioid agonist therapy
OMHSP: Office of Mental Health and Suicide Prevention
OUD: opioid use disorder
PCMSS-MAT: Providers’ Clinical Support System for Medication Assisted Treatment
PDSI: Psychotropic Drug Safety Initiative
SUD: substance use disorder
VA: Department of Veterans Affairs
VHA: Veterans Health Administration
